# An embedded multiple case study: using CFIR to map clinical food security screening constructs for the development of primary care practice guidelines

**DOI:** 10.1186/s12889-021-12407-y

**Published:** 2022-01-14

**Authors:** Sabira Taher, Naoko Muramatsu, Angela Odoms-Young, Nadine Peacock, C. Fagen Michael, K. Suh Courtney

**Affiliations:** 1 Department of Preventive Medicine, Northwestern Feinberg School of Medicine, 680 N Lake Shore Drive Suite 1400, Chicago, IL 60611 USA; 2 Department of Community Health Sciences, School of Public Health, University of Illinois at Chicago, 1603 W. Taylor Street, Chicago, IL 60612 USA; 3 Division of Nutritional Sciences, College of Human Ecology, Cornell University, Martha Van Rensselaer Hall, Ithaca, NY 14853 USA; 4 Department of Family Medicine, Loyola Stritch School of Medicine, 2160 S 1st Ave, Maywood, IL 60153 USA

**Keywords:** Food insecurity, Food security screening, Implementation, Dissemination, Consolidated framework for implementation research, Primary care practice, Semi-structured interviews, Produce prescription programs

## Abstract

**Background:**

Food insecurity (FI), the limited access to healthy food to live an active and healthy life, is a social determinant of health linked to poor dietary health and difficulty with disease management in the United States (U.S.). Healthcare experts support the adoption of validated screening tools within primary care practice to identify and connect FI patients to healthy and affordable food resources. Yet, a lack of standard practices limits uptake. The purpose of this study was to understand program processes and outcomes of primary care focused FI screening initiatives that may guide wide-scale program implementation.

**Methods:**

This was an embedded multiple case study of two primary care-focused initiatives implemented in two diverse health systems in Chicago and Suburban Cook County that routinely screened patients for FI and referred them to onsite food assistance programs. The Consolidated Framework for Implementation Research and an iterative process were used to collect/analyze qualitative data through semi-structured interviews with *N* = 19 healthcare staff. Intended program activities, outcomes, actors, implementation barriers/facilitators and overarching implementation themes were identified as a part of a cross-case analysis.

**Results:**

Programs outcomes included: the number of patients screened, identified as FI and that participated in the onsite food assistance program. Study participants reported limited internal resources as implementation barriers for program activities. The implementation climate that leveraged the strength of community collaborations and aligned internal, implementation climate were critical facilitators that contributed to the flexibility of program activities that were tailored to fill gaps in resources and meet patient and clinician needs.

**Conclusion:**

Highly adaptable programs and the healthcare context enhanced implementation feasibility across settings. These characteristics can support program uptake in other settings, but should be used with caution to preserve program fidelity. A foundational model for the development and testing of standard clinical practice was the product of this study.

**Supplementary Information:**

The online version contains supplementary material available at 10.1186/s12889-021-12407-y.

## Background

### Food insecurity

Food insecurity (hereafter FI) is a social determinant of health and economic condition where limited access to safe, high-quality, nutritious food prevents individuals from leading active and healthy lives [1]. The World Bank estimated that acute food insecurity increased drastically and on a global scale due to COVID-19, and that the majority of these cases were connected to hunger in International Development Association countries driven by climate change, long-lasting conflict, and other economic conditions [2]. Low levels of education and limited social networks were consistent variables across countries that increased the risk for FI. Yet, country-specific economic, political and sociocultural factors varied greatly between countries, which highlights the need to utilize country-specific interventions and policies to reduce FI on a local level [3]. This study specifically focuses on FI and the healthcare context in the U.S., which, when compared to peer nations, has the highest number of preventable chronic illnesses related to poor nutrition, as well as hospitalizations and deaths [4].

Prior to the COVID-19 pandemic, approximately 33.5 million Americans experienced FI [1]. Since April 2020, that number has nearly doubled [5].. FI in the U.S. is characterized by the overconsumption of poor quality, highly processed, calorie dense food that is extremely affordable and widely available [6]. U.S. households most affected by FI are low-income, ethnic and minority communities, especially those affected by unemployment and job loss [7] FI contributes to the limited ability to eat a healthy diet, often the first recommended step for disease management. Thus FI contributes to the high prevalence of obesity, diabetes, cardiovascular disease, and difficulty with disease management among low-income U.S. populations [6].

### Recommendations for screening and linking patients

Studies show that local and federal U.S. food assistance services remain underutilized due to limited awareness about their existence, the stigma associated with using welfare programs, and complex enrollment processes that can discourage use [1, 11–13]. There is a growing body of evidence that illustrates how partnerships between healthcare systems and local food assistance programs can increase the use of services and help improve dietary health [8–10]. Research shows that recommendations for identifying FI patients through routine FI screening with the validated Hunger Vital Signs™ tool, and referring patients to evidence-based programs, such the Supplemental Nutrition Assistance Program (hereafter SNAP) and community food pantries, increases the use of food assistance services, and has demonstrated immediate dietary improvements in cancer, diabetes and hypertensive patients [11–15].

### Addressing food insecurity in primary care practice

Among screening initiatives that exist, those implemented in primary care settings demonstrate the most potential to address FI because primary care practice is the most common form of health care delivery in the U.S. Moreover, primary care is recognized by professional and government healthcare organizations as the most typical setting where referrals to social and community resources often occur that connect patients to basic needs for disease management [8, 16–18].

### Lack of evidence that points to improved health outcomes

Existing literature points to FI screening practices that are largely guided by broad, national principles that have been interpreted in many ways—perhaps due to their rapid and organic evolution to fill a growing FI crisis. Evidence suggests that program activities, actors, implementation processes are driven by each healthcare contexts, program outcomes vary across clinical settings, and as a result, the long-term impact on health outcomes of screening programs cannot be determined [9, 19, 20]. The challenge stems from the lack of translational research and rigorously tested standard practices in this relatively new area of clinical practice [20, 21]. The gaps in the literature suggest that we need to examine how these programs operate in real-world settings, and identify which program activities have the most potential for generalization. This can inform the development of practice guidelines that can be tested in effectiveness trials, and eventually implemented and tested on a wider scale.

### Implementation science

In implementation science, theory derived frameworks are used to study implementation context—specifically how multilevel system wide factors (e.g. individual level organizational level) and multisector factors (i.e. policies, external partnerships and community needs) interact and determine the quality of implementation outcomes [22]. Findings from implementation science studies allow researchers to hypothesize the relationship between implementation factors. These contextual variables can be tested in other settings where program adaptations maybe considered that lend themselves to wide-scale dissemination of evidence-based practice [22].

Implementation science research has been supported in several healthcare research studies, most notably by the National Institutes of Health for a variety of social and behavioral health research, such as tobacco cessation and diabetes prevention. The purpose of these studies was to understand how the complex and interdependent sociocultural, economic and political factors within the implementation context affected the process of program implementation. There is an opportunity to apply implementation science in the context of clinical FI initiatives because theoretical underpinnings of implementation have not yet been explored. Ultimately, researchers used findings to determine how to effectively disseminate and adapt these programs into other healthcare settings and contexts [22, 23].

### Consolidated framework for implementation research

The Consolidated Framework for Implementation Research (hereafter CFIR) is an Implementation determinants framework comprised of theoretically derived domains and constructs as seen in Table 1. CFIR has been empirically tested and is widely used in healthcare settings to understand multidimensional, interrelated implementation barriers and facilitators within specific healthcare organizations [24–26].

**Table 1:** Characteristics of study cases

Cases	Characteristic of healthcare organization	Program funding	Food organization	Location	Initiative	Stage of implementation
1. Program A	Public, government funded healthcare system	None	Local food bank	Urban setting	Food security screening, mobile food truck, enrollment/referral to benefits program	Full Implementation (1 year and beyond)
2. Program B	Private, academic medical center	Federal funding and local grants	Urban garden collective	Suburban setting	Food security screening, onsite food distribution, enrollment to benefits program	Full Implementation (1 year and beyond)

The framework is broad with over 30 theoretically-derived constructs. When used to map implementation factors, CFIR can help researchers establish a foundation from which semantic relationships between implementation factors can be constructed. Hypothesized relationships between constructs can be used to develop a conceptual model that describes the implementation of a specific intervention. The framework is made up of implementation drivers that are categorized into five domains: 1) The intervention characteristics that point to the quality of the program, compatible design, its cost and adaptability across settings. 2) The inner setting, which directly relates to the physical and cultural setting where daily program processes occur. 3) The outer setting, which refers to any factor external to the program itself, including community needs, influences, local mandates, policies or regulations that affect implementation processes. 4) Characteristics of program staff/individuals, which are their knowledge and beliefs about the program from their own perspective. 5) Implementation processes, which include the steps used in planning, execution and ongoing management of the program [22].

The purpose of this study was to understand implementation processes and outcomes of two distinctively different FI screening initiatives. One program was implemented in primary care clinics located within the context of an urban, government funded health system. The other program was implemented in primary care clinics associated with a suburban, private, academic medical center. A total of *N* = 19 healthcare staff participated in one-on-one interviews in this study to provide their perspectives about implementation. We used the Consolidated Framework for Implementation Research (CFIR) to identify common implementation barriers and facilitators from each interview as a part of a cross-case analysis. Findings from this study were used to develop a formative conceptual model that can guide the development, refinement and testing of standard screening practices in future research.

## Methods

### Selection of study cases

Cook County is located in Illinois, a state located in the Midwestern region of the U.S. Within Cook County is a complex healthcare network that serves 5.2 million people. Two FI screening and referral programs were selected for this study using criterion sampling from a larger sample of 13 programs implemented within primary care settings identified in a previous study. The two programs selected for this study (hereafter Program A and Program B) differed in the type of setting (i.e. one public, government funded organization, the other an academic medical center). Distinct program differences listed in Table 1 allowed for the exploration of program implementation in different contexts and the extraction of common, overarching implementation themes.

Inclusion criteria were based on previous research and national recommendations for clinical FI screening initiatives [8, 17, 18, 27]. Study cases met the following criteria: 1) Programs that utilized the standardized two question Hunger Vital Signs tool to screen patients for FI; 2) Programs that incorporated a referral system to onsite food services for FI patients; 3) Programs that incorporated a referral system for FI to enroll in SNAP and other federal benefits; 4) Programs that had been implemented for a minimum of one year. The last criterion allowed for the examination of programs that had been presumably functioning long enough that initial challenges common to start-up programs had already been addressed.

### Study design

**Fig. 1:**
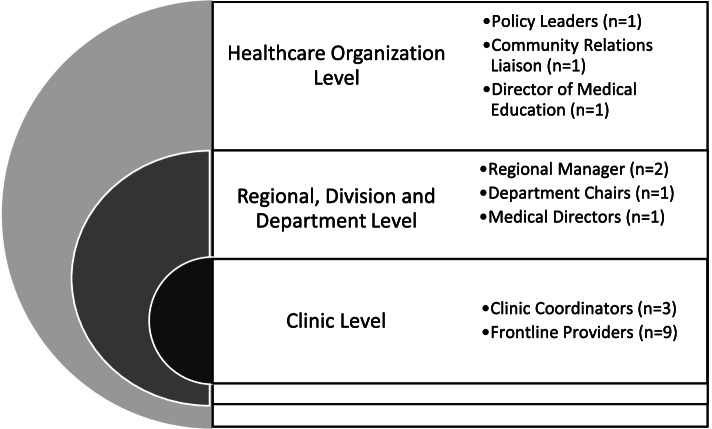
Units of analysis across healthcare organizations in this study (*N* = 19)

An embedded multiple case study design was used to examine the phenomenon of primary care situated FI screening and referral processes [28, 29]. The embedded nature of this study refers to the multiple units of analysis within each case [29]. Preliminary research for this study indicated that the healthcare context (e.g. clinicians at the practice level) drove how FI screening programs were implemented and what types of food assistance programs were incorporated for referral. Therefore, each case in this study was identified as one individual screening initiative and the units of analysis were clinical program actors within the healthcare setting as illustrated in Fig. 1.

### Participants

From September 2019 to March 2020, an iterative sampling approach was used to recruit participants for this study from a convenience sample of implementation actors at each case until data saturation was achieved (N = 19). Through a purposive sampling process, implementation leaders, clinicians and other healthcare staff critical to program implementation were recruited for this study [30].

### Study instrument and data collection

The interview guides used with organizational leaders and frontline providers were developed for this study using the adapted CFIR framework (available in Additional File 1 “Interview Guide for Key Program Planners” and “Frontline Provider Interview Guide”). As in similar research, interview questions broadly asked about program activities, implementation processes, program outcomes and asked participants to identify major challenges/facilitators that affected feasibility and fidelity of program implementation [31].

A trained qualitative researcher (ST) conducted semi-structured, key informant interviews for this study. The interviews were conducted face-to-face at each program site or over the telephone at the study participant’s discretion. Each interview lasted 30–45 min and were audio recorded for data analysis purposes. Participants recruited for the study were made aware of the audio recording at the beginning of each session and were required to provide verbal consent prior to participation in the study. This study and the verbal consent process were approved for a claim of exemption (Protocol # 2019–0610) from the University of Illinois at Chicago Office for the Protection of Research Subjects Institutional Review Board on August 30, 2019.

The researcher took detailed notes during each interview that provided initial insights to the study. Revisions to the instrument guide were made after each interview for clarity and to collect additional program details.

### Data collection, coding and analysis

**Fig. 2:**
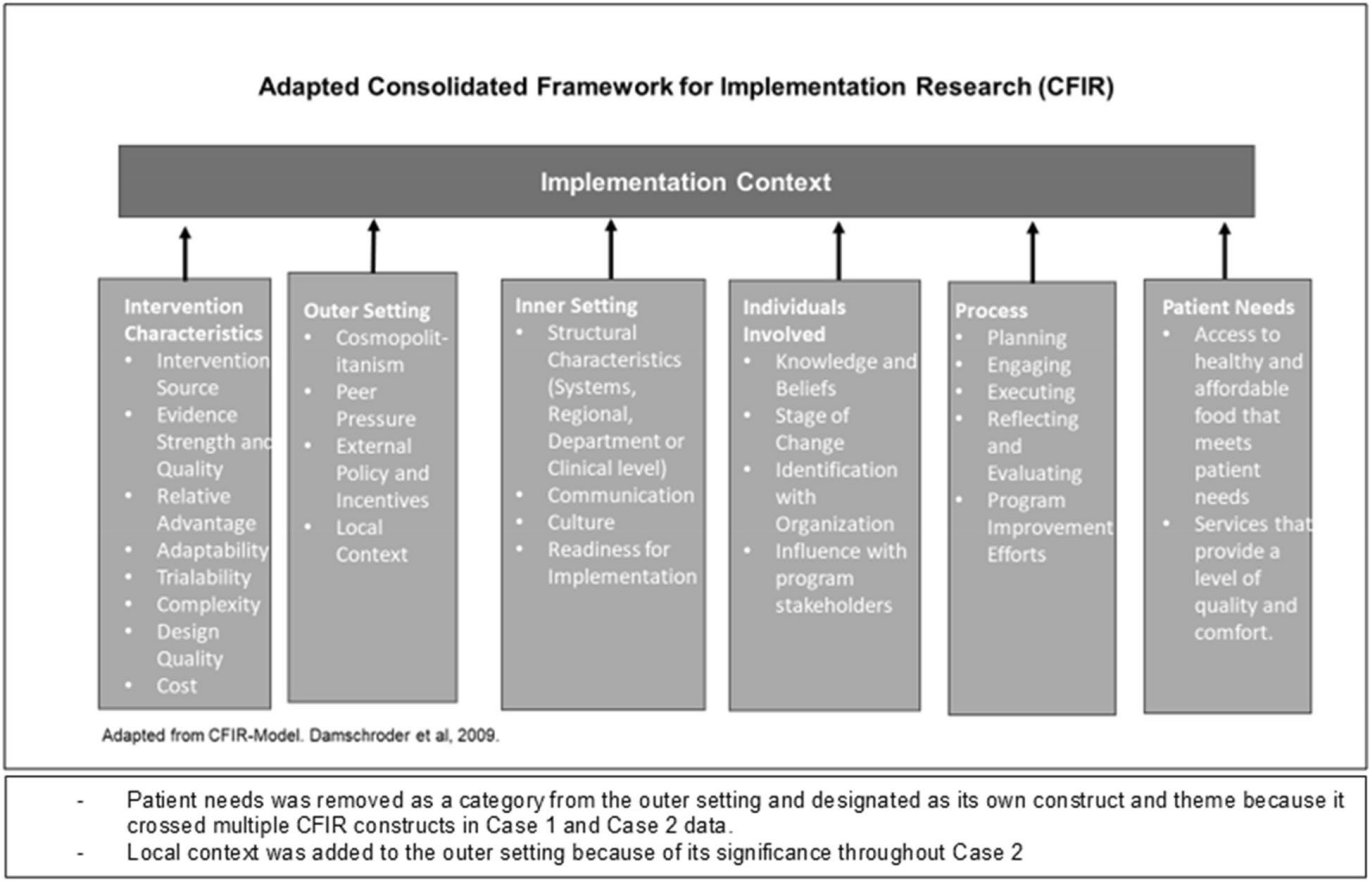
Adapted CFIR framework for this study

Data were collected, managed and analyzed concurrently over a period of seven months until data saturation was achieved. Transcriptions of the interviews were uploaded to Atlas.ti v.8 Qualitative Data Analysis Software for data management, coding and assistance with analysis. All personal identifying information was removed from the data prior to analysis. All data were stored on a password protected computer only accessible by the researcher. A codebook developed a priori based on the adapted CFIR framework for data interpretation was used during data collection and analysis (see Additional File 2, “Study Codebook”). Codes were added to or removed from the codebook based on previous research, organizational theory, and as new ideas and concepts emerged, illustrated in Fig. 2 [9, 32, 33].

Two experienced PhD level university students (ST and LC) established interrater reliability of the coding process until 80% agreement was achieved as recommended for qualitative research [34]. As data were collected, memos were used to document progress, study decisions and emerging themes [35]. Matrices and frameworks were developed to guide thematic analysis and anchor emerging concepts to specific CFIR constructs [36]. The themes and patterns that emerged from each interview were compared to previous interview findings. This allowed the identification of commonalities, disparities and outliers in the data and for a rich understanding of program implementation to emerge [28].

For each case, program activities, time of occurrence and implementation actors were confirmed. Implementation processes were also described as originally intended, as well as unanticipated implementation facilitators and challenges and the unique implementation context that resulted in program adaptations.

Program outcomes were also collected to assess implementation feasibility, effectiveness, as well as overall program fidelity [31, 37]. The following program outcomes were identified across cases: the number of patients screened; the number that identified as FI; the number of patients referred to food assistance programs; the number of patients that participated in the food assistance program. The frequency that clinicians completed essential program activities was also collected to tie outcomes to specific program elements. During the cross-case analysis, the binding implementation themes were identified and gave meaning to program outcomes.

Atlas.ti v8 exploratory functions were used to further analyze and confirm findings, and for source triangulation between participants. Any overlap of themes helped to establish the semantic relationships between CFIR constructs. Prior to the finalization of study results, one program leader and one clinician from each case were asked to participate in member-checks. They each reviewed the results from their respective case, and provided feedback where necessary to ensure validity of study findings.

## Results

### Program activities and process outcomes

Study findings revealed similar intended program activities and processes within and across cases illustrated in Tables 2 and 3.

**Table 2:** Description of intended activities and actors of each program

Case	Intended program activity	Intended implementation actor
Program A	1. EHR FI screening once a year during patient intake	Medical Assistant
	2. Refer FI patients a fresh produce truck during doctor’s visit with a voucher to receive free produce	Physician
	3. Referral to local food assistance resource list during doctor’s visit	Physician
	4. SNAP enrollment right after doctor’s visit	Social Worker
	5. Phone call reminder to voucher recipients one to two weeks prior to the day of food distribution	Social Worker
	6. Food distribution once every two months through a fresh produce truck parked outside clinic	Social Worker in collaboration with food partner
	7. Evaluation by collecting food truck participation rates through voucher redemption	Social Worker
Program B	1. Screening during collection of patient vitals during routine doctor’s visit; positive screen flagged in the EHR	Nurse or Medical Assistant
	2. Referral to local food assistance resource list during doctor’s visit	Physician
	3. Referral to produce prescription program	Physician
	4. Call FI to enroll in the produce prescription program	Program Manager
	5. The Produce Prescription Program was held weekly during a two-hour window, one evening per week that included nutrition education classes	Program Manager
	6. SNAP eligible patients could enroll in SNAP using an electronic tablet provided by the clinic	Program Manager
	7. A weekly patient satisfaction survey distributed to patients after program participation. Questions asked about food preferences, cooking and nutrition lessons. Every fifth session clinical staff distributed a survey to measure change in FI status or improvements in dietary behavior due to program participation.	Program Manager

**Table 3:** Process outcomes

Program activity	Program A		Program B	
	Number of Patients*	Proportion of patient population	Number of Patients**	Proportion of patient population
Intended number of patients screened for FI (patient population served per month)	1250	100%	453	100%
Actual number of patients screened for FI	1250	100%	326	72%
Number of patients screened positive for FI	300	24%	140	31%
Number of patients referred to the food assistance intervention	300	24%	140	31%
Number of patients received resource list	Data unavailable	Data unavailable	Data unavailable	Data Unavailable
Number of Social Worker visits to manage FI	Data unavailable	Data unavailable	N/A	N/A
Number of FI patients identified through screening that received phone call reminders	100	8%	100	22%
Number of patients that participated in the food assistance intervention	167	13%	115	25%
Number of patients enrolled in SNAP benefits	0	0	0	0

*Because Program A included 3 study clinics, outcomes were averaged across the three study clinics at one point in time. Program A tracked program activities from the start to the end of each month

**Program B reported data cumulatively over the course of the 6 month program. At the time data were collected the

Both cases intended to screen all of their patients for FI using their EMR systems and projected that 45% of their patient population would be identified as FI based on previous community-wide data [38]. Across cases, screening took place by a clinician approximately one time a year prior to the doctor’s visit during intake or in the clinic waiting room. Positive responses were documented and flagged in the patient’s medical record to prompt the physician to discuss FI during the patient visit. Between 24 and 31% of patients were actually identified through screening and were referred to an onsite food distribution program that provided fresh produce at no cost to FI patients. During the doctor’s visit, all patients identified as FI were projected to receive information or list about local food assistance resources and should have been referred to the Social Worker to enroll in SNAP benefits if eligible. These data were either unavailable for this study or this program activity was not performed.

Clinicians across cases intended to use phone call reminders as an opportunity to remind and educate FI patients about the benefits of participating in the food distribution programs. Between 8 and 22% of patients received phone call reminders.

### Thematic analysis findings

**Fig. 3:**
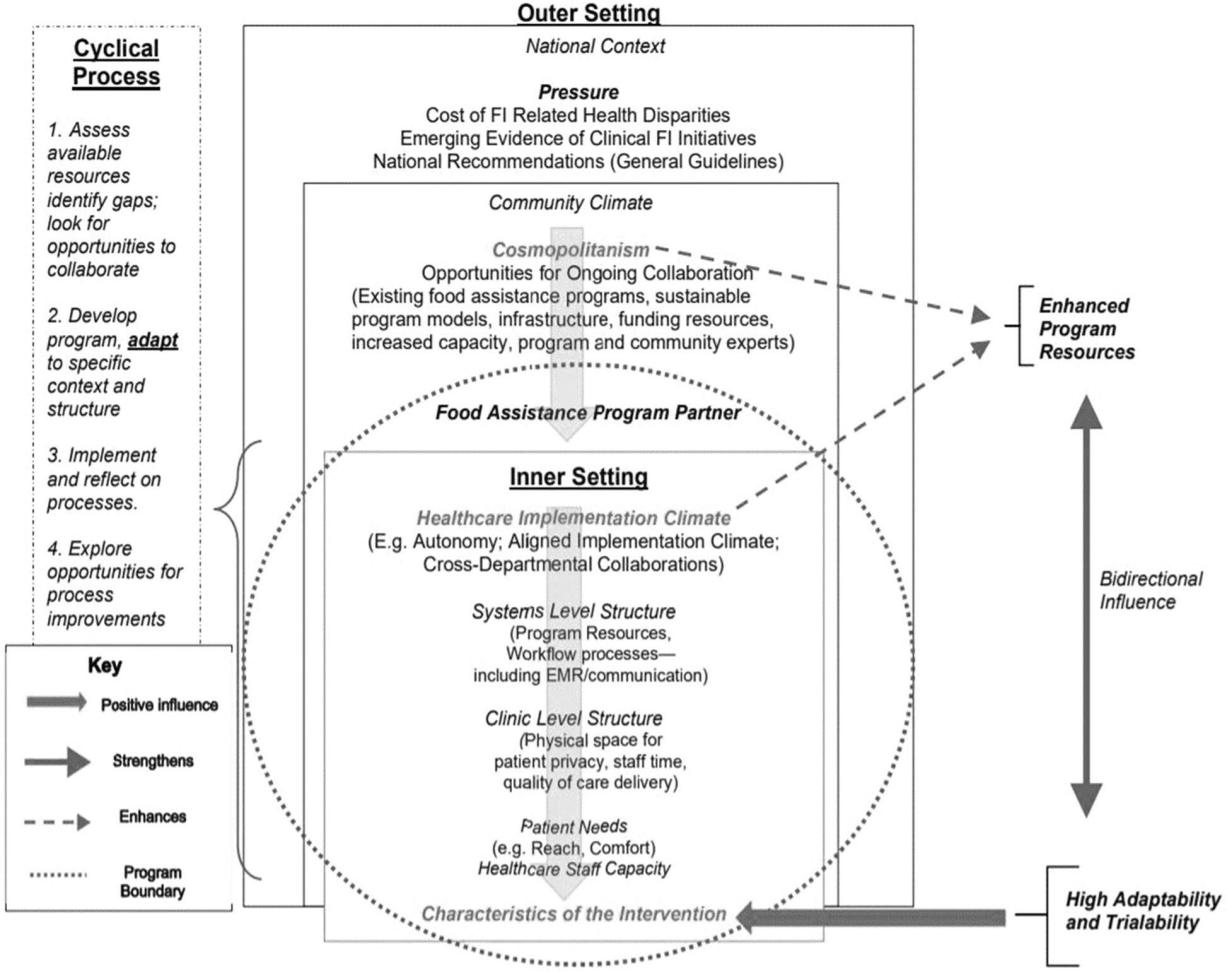
Formative conceptual model for implementation

Figure 3 represents a formative conceptual model utilizing CFIR concepts that sums up overarching themes described below. The model illustrates the semantic relationships between core CFIR concepts revealed during thematic analysis that helps to explain why process outcomes were lower than projected.

### Barriers within the inner setting

Clinicians across cases reported that physical space, clinician capacity, financial resources and EMR technology were resource challenges that inhibited program implementation. The study revealed a hierarchical relationship where barriers study participants identified trickled down from the organizational systems level that resulted in challenges with the delivery of care at the clinic level and ultimately affected how patients experienced FI screening and referral processes as seen in Fig. 3. Identified in similar intervention studies, these challenges speak to the broader applicability of study findings to other U.S. healthcare settings [39].

Study participants agreed that due to their health system’s pre-programmed EMR software, clinician prompts for FI screening and resource lists were not functional for the realities of day-to-day clinical care. Study participants reported that electronic screening tools were either unavailable or were only available intermittently (e.g. once per year), which did not allow for clinicians to capture the episodic and cyclical nature of FI. Resource lists were embedded so deeply within the EMR system that study participants also reported that they did not have time to navigate to those lists during patient visits.

Limited financial resources that health systems could allocate to screening and referral initiatives negatively affected the frequency of food distribution, program sustainability and reach. Study participants reported that screening and referral programs were supported by a finite amount of in-kind donations from their community food assistance partner and local and federal grants. As a result, funding would last for only a fixed amount of time, and study participants reported that the money just did not pay for enough food meet patient needs. Moreover, grant dollars had spending restrictions, and required patients to be enrolled in SNAP benefits. Study participants reported that because of this spending rule, they had to turn several of their low-income patients away during program activities if they were not eligible for SNAP benefits, which negatively affected program reach.

Rigid workflow processes across cases provided a small window of opportunity for FI screening and referral during the patient visit. Study participants said that check-in and intake were activities that occurred in settings with very little privacy, but indicated that this was typical for how clinic waiting room or nurse’s intake stations functioned. Physical space became a barrier to screening, referral and food distribution. Across cases, patients displayed discomfort when presented with FI questions, which study participants believed was because of the lack of privacy from other patients. Some patients commonly denied experiencing FI, even if clinicians knew that this was not the case. A lack of privacy could explain why a lower than expected number of patients were identified as FI, and were referred to and participated in the food distribution programs.

Study participants reported that clinician capacity to deliver patient care was dependent on the health system’s workflow, and consistently reported that its rigidity did not provide enough time to distribute food assistance resource lists and counsel patients about FI. Clinicians also reported the inability to conduct patient outreach and phone calls for the purpose of increasing awareness about when food distribution was scheduled—an important component for increasing program reach. This finding could explain why there were lower than expected number of patients participated in the food distribution programs overall.

### Leveraging the implementation context as a facilitator

Across cases, study participants reported that the support of community partnerships and the internal work culture created an aligned implementation context. As seen in Fig. 3, the community climate—cosmopolitanism—played a big role during program planning and execution and clinic-level autonomy allowed clinicians to make timely program adaptations when faced with resource challenges.

Multi-sector networks supported inherent synergy with existing community initiatives, which gave each health system access to an existing program model, expertise and infrastructure. The local food justice organization and food depository that partnered with the health systems advocated for an equitable and sustainable food system through existing initiatives throughout the community and played a fundamental role in bringing together health systems, local food growers and other health and wellness community organizations; the collective strength and presence of which created a supportive implementation climate where study participants reported an activation of knowledge, awareness and advocacy work among clinical staff in preparation for program implementation.

For example, study participants reported the presence of farm stands across the health system campus, medical and dietetics students involved in program activities, the use of offsite community centers to increase program participation and the assistance of local grant opportunities to increase food production and reach.

A culture of clinic-level autonomy facilitated the equitable distribution of decision-making authority. “Managers need to take ownership because they know their patients and their staff,” said one study participant and in turn, when asked about this, clinicians responded with, “We do things differently here,” and “the way we do it is we want to reach everyone…” Statements of this kind referred to linkages, voucher activities and food distribution processes that study participants reported were adapted for universal distribution to reduce the stigma of FI. This adaptation could explain why the number of patients that participated in food distribution programs were much greater than the number of phone call reminders, and SNAP enrollment activities that clinicians actually engaged in.

### Adaptability and trialability of program characteristics

Study participants reported that program activities were highly adaptable and testable. Adaptability refers to the degree to which the core program components can be tailored to fit the implementation context [22]. Trialability refers to the ability for stakeholders to pilot an intervention on a small scale and engaged in quality improvement efforts [22]. As seen in Fig. 3, both constructs emerged in the context of limited program resources. It appeared that when limited program resources were supplemented, high levels of adaptability and trialability characteristics were revealed. Conversely, the high level of adaptability and trialability of each program allowed for ongoing exploration of alternative and creative methods to improve program implementation, reach and sustainability, suggesting a bi-directional relationship between these program characteristics.

Table 4 lists the overarching study themes and illustrative quotes that assisted in the interpretation of findings.

**Table 4:** Overarching study themes and illustrative quotes

Theme	Illustrative quotation
Limited healthcare resources/EMR	“IT has not been very helpful because they have not been involved with our program so we’re still using paper surveys…we only have one person in charge of screening and she’s overwhelmed…” –Clinician, Program B
	“Screening for food insecurity once a year isn’t enough, but that’s how our EHR works.” Clinician, Program A
Limited healthcare resources/EMR	“The food insecurity questions are not embedded in the EHR…the paper system is inefficient for assessing food insecurity needs across the board.” --Clinician, Program B
Limited healthcare resources/space	“Unfortunately, it’s (screening) not very private. And what I mean by that is that it’s open to more medical assistants that are sitting at that station and potentially another patient getting vitals next to them…So as they’re asking them the questions, there are more people around and it’s not very private. Sometimes we do have that response of no, no, no I’m fine and then they get inside and they tell the doctor maybe something different.” –Clinician, Program A
	“It’s up to the clinic staff to follow through with how they do this because we respect the autonomy of each clinic to take responsibility for the program and how it’s done.” –Program Leader, Program A
Leveraging context (clinic-level autonomy) to foster implementation adaptations	“Yeah, so anyone can get a voucher that’s a patient. How we do it is that even after the nurses give them out, we give vouchers to the clerks and other staff that may run into patients. A patient may come up just to pick up some documentation and be like, ‘Oh, I know the Fresh Food Truck has come in. Can I have a voucher?’ Everyone has access to the vouchers so they can give them out at any time on any day.” –Clinician, Program A
	“In general, food insecurity has not been on my radar... As you probably know, a primary care visit is completely overwhelming with—'I have to get your foot exam done, you have to get an influenza shot, I've got to draw your A1C.’ Food insecurity is a serious conversation that takes time…And so that is why it has not been a part of my standard practice.” – Clinician, Program A
	“We don’t have a way of flagging patients that are food insecure in the EHR to remind doctor’s to talk to patients about resources and sometimes they forget” –Program Leader, Program A
Limited healthcare resources/staff capacity	“So we used to have a [phone call] list of the patients who answered yes to the food insecurity questions, but [the phone call reminders] didn't work with us, and was very ineffective because a lot of times patients say no [when asked if they experience food insecurity], but it’s really yes. So their name wouldn't be on the list. So we just wiped out the list altogether and emphasize ‘don't lose your voucher.’” –Clinician, Program A
Limited healthcare resource/EMR	“When we did it in the summer here at the clinic, space is definitely limited…you can imagine how tight it was… I think our partnership with the park district and their marketing efforts really drew many of the participants. I think that partnership is really, was one of the keys to our success. They shared it on their Facebook page and literally like the next week we had double the amount of participants” –Clinician, Program B
Leveraging context (clinic-level autonomy) to foster implementation adaptations	
Limited healthcare resources/space	
Leveraging context (existing community partnerships and growing model) to foster program reach, acceptability and support	
Limited healthcare resources/financial	“The food truck visits at each site are not frequent enough because it requires an incredible amount of [food pantry] capacity to staff to run the truck and we don’t have the funds to pay for that. Bad weather, like last year’s polar vortex, can deter participants from coming to the distribution…Patients want more frequent distribution.” –Program Leader, Program A
Limited healthcare resources/financial	“On that [USDA] grant it’s a total of four [years]…I think we’ve also realized that it’s not a sustainable solution to food insecurity. We’re really trying to think of what else can we do to make this a sustainable food economy here in [neighborhood name].” – Program Leader, Program B
Leveraging context (existing community partnerships and growing model) to foster program acceptability and support	“We’ve been close partners with [the urban garden collective], and we wrote the USDA grant together for [Program B]. So, the growing concept came from them…One, the hospital alone can’t do it, but when we all came together it was feasible.” –Program Leader, Program B
Leveraging implementation climate (existing growing model) to foster program acceptability	“I think the strength of having it at the clinic is just the traffic. The traffic of people. So, people walking past the farm stand and seeing the vegetable distribution get naturally brought into the cooking demos… the farmstand is a brilliant move because that farmstand is sitting there just seamlessly with the [prescription produce box] distribution… It becomes a very collective experience. “–Clinician, Program B

## Discussion

This is the first study, to this author’s knowledge, that applied CFIR to examine system-wide implementation factors of clinical FI screening initiatives within the context of healthcare settings and primary care. Empirically tested and theoretically derived CFIR concepts guided the development of a conceptual implementation model, while integrating program outcomes strengthened the interpretation of qualitative findings. The conceptual model in Fig. 3 may be tested and refined in follow-up studies to facilitate implementation and increase program reach, impact and sustainability.

In this early stage of formative research, one optimal combination of clinical screening and referral activities did not emerge as generalizable for testing on a larger scale, which is a necessary step in the translational research pipeline [40]. The U.S. healthcare system’s fragmented payer system, lack of universal coverage and disparities in cost and quality of healthcare may have contributed to this finding, and was reflective in this study when each program was operationalized in different ways to meet the unique challenges, needs and context of each health system and patient population.

Nevertheless, overarching themes that emerged across cases that maybe generalizable. Salient to this study were the CFIR concepts, program adaptability and trialability that made implementation feasible across both cases, while maintaining core screening and referral activities. This is consistent with the scalability and implementation framework literature that relies on assessing context, such as available human capital, technical resources, financial costs, and any other contextual factors that may not be replicable in a larger study, but that provide information about the authenticity and feasibility of delivering core intervention activities in clinical practice [26, 40, 41]. This finding is also reflective in and policy recommendations for SDOH screening practices that identify the flexibility of SDOH screening program activities to meet the health system context, including patient and staff needs [42, 43].

Building on this concept, the proactive support of intervention modifications has been proposed in emerging health equity research as a way to address disparities in healthcare delivery, access, resources and outcomes in our most vulnerable populations [44]. It requires the documentation of intervention modifications, which enhance fit or effectiveness in a given context that can lead to improved engagement, acceptability and clinical outcomes [44, 45]. Documentation of key adaptations can also facilitate more rigorous feasibility studies when researchers clarify the context of adaptations, such as the reasoning, timing, and process of modifications that facilitated implementation, scale-up, spread or sustainability, and should be considered in future clinical FI screening research that builds on this study [45].

Moreover, adaptability and trialability highlighted the significance of the CFIR cosmopolitanism concept in this study. Specifically, the interaction between the inner culture and community context drove program design and filled healthcare resource gaps. This finding reflects the current literature on the existences of clinical-community linkages to address FI through clinical screening and referral mechanisms [8, 9]. It also points to a multi-sector response that has already demonstrated effective collaborations between primary care and community organizations in the control and management of communicable and chronic diseases by establishing a medical home that is patient and community centered [46, 47].

## Recommendations

Study findings resulted in the following recommendations for health systems: 1) Allow for adaptations with caution. Unique implementation contexts can foster implementation feasibility. Yet, considerations need to be made about how adaptations may negatively impact fidelity, reach and effectiveness. 2) Consider how the context can support intervention activities through clinician input about workflow, program responsibilities and time management. 3) Conduct asset mapping and outreach to potential community partners that have a strong presence in the community, aligned goals and objectives and resources that can be leveraged during program design and implementation. This recommendation raises its own challenges about whose responsibility within the health system it is to make community-wide connections and manage relationships, but is key for establishing a truly patient and community-centered medical home. 4) Consider non-traditional forms of staff support. In this study, allied health and medical students were motivated to work as interns in exchange for hands-on, experiential learning. Generally, students are subject to high turnover and may not always be the best solution to fill staffing shortages that require a long-term commitment. An alternative solution is to leverage the role and expertise of community health workers that are trusted sources of information for patients because they often live within the communities they serve.

## Limitations

1. As a study instrument, the researcher was positioned alongside study participants during the process of information discovery during data collection and analysis. As such, this was a subjective process that may have been affected by the researcher’s own biases and experiences [48]. The researcher utilized source triangulation and member checks to negate the effect of these factors during data analysis and interpretation.
2. While this study incorporated the perspective of multiple implementation actors representative of the implementation context, the sample size may be considered small at first glance. What is important to note is that data saturation was achieved, and that qualitative research of this nature requires the deep exploration of the context to interpret findings in a meaningful way. The scope of the study may have been expanded to incorporate more programs and program staff if time and resources to complete this study had not been limited.
3. The study did not include patients’ perspectives or in vivo observations of screening and referral processes. Real-time data could have enhanced study findings, and patients’ perspectives could have provided insight about how screening and referral processes affected their clinical experience. The amount of time allotted for this study limited the scope of the study to the perspective of implementation actors only. Moreover, due to patient privacy laws, the study sites would not allow researchers to sit in during clinical visits. Future studies should consider patient interviews and immediate, post visit surveys to gauge a patient’s perspective about screening and referral processes.
4. Due to time restrictions, data that were collected at only one point in time and relied on the memory of each participant. Future studies should consider the collection of data from participants at multiple time points to capture the dynamic process of implementation and to further validate findings.
5. Lastly, this study is applicable only to the context of the U.S. healthcare system and characteristics of FI within the U.S. Nevertheless, a community-clinical integrated model may have the potential to address hunger in other countries.

## Implications

This study makes significant contributions to the limited body of literature in the emerging field of clinical FI screening programs in primary care practice. In particular, the proposed conceptual model is a foundation for the development of theory-driven standard practices. Though formative in nature the model identifies areas of exploration that have not been considered in previous research, such as intervention adaptability, internal work culture and the community climate.

Study findings have implications for practice-based research. The exploration of external factors and creative uses of internal assets for program support should be considered due to the scarcity of funding for community-based interventions implemented in low-resource clinics. Future work should consider how these factors may enhance limited internal resources long-term. Community-engaged formative research with patients could help to tailor primary care focused initiatives to the realities of patient needs. Engaging the patient community could provide critical insights about stigma, privacy, trust and workflow processes from the patient’s perspective, as well as provide deeper understanding about the cyclical nature of household FI that may inform frequency of screening and can be used to advocate for additional health services. Study findings also have implications for ongoing policy work of universal social determinants of health screening practices supported by national healthcare experts.

## Conclusion

The key take away from this study is that due to limited healthcare resources, primary care practices that serve low-income communities need to be supported in their ability to adapt program activities to their specific context. While high program fidelity and intended program outcomes may not have been achieved in this study, findings demonstrate how implementation feasibility can be achieved when community partnerships and an internal resources are leveraged for program adaptations and support. With this in mind, future research may continue to build on the proposed conceptual model, which is formative in nature and sets the stage for development of standard screening practices. As our healthcare system continues its transition to a value-based model of care, we need to consider how primary care focused FI screening initiatives can effectively connect patients to food resources. If we can reduce the inequitable access to affordable and healthy food, we may eventually see long-term improvements in the quality of life of our most vulnerable populations.

## Supplementary Information

**Additional file 1.**

**Additional file 2.**

## Abbreviations

FI: Food Insecurity/Food Insecure
U.S.: United States
SNAP: Supplemental Nutrition Assistance Program
WIC: Special Supplemental Nutrition Assistance Program for Women’s Infants and Children
EHR: Electronic Health Records
EMR: Electronic Medical Records
CFIR: Consolidated Framework for Implementation Research
AHE: Alliance for Health Equity
