# Supplementary material for: An embedded multiple case study: using CFIR to map clinical food security screening constructs for the development of primary care practice guidelines
Source: BMC Public Health. 2022 Jan 14;22:97. doi: 10.1186/s12889-021-12407-y (PMC8758892; doi:10.1186/s12889-021-12407-y)
Supplement: Supplementary file 1 — Additional file 1. [file 12889_2021_12407_MOESM1_ESM.docx]

**Interview Guide for Key Program Planners**

**INTRODUCTION:**

I’m conducting interviews with key individuals from those organizations that have a focus on screening for food insecurity in primary care settings. I want to learn more about the planning stage, program features, implementation processes, individuals involved in implementation and what barriers and facilitators you have experienced during the stages of program planning and implementation.

The interview will be used to understand how organizations develop partnerships with food access organizations to address food insecurity and the process for program implementation. Your participation in this interview is important because it will inform the development of program guidelines that may be distributed to other healthcare organizations interested in implementing a similar initiative.

Do you have any questions before we begin?

1. How would you define food insecurity or low food security?
2. What is your role or job title in this healthcare organization?
3. What is the name of the initiative we are discussing today and how long has your program been operating?
4. What is your role in implementing the (Program Name)?
5. How were you selected for this role?

***Probe:*** Were you assigned by senior staff, did you volunteer, were you nominated?

***Probe:*** What knowledge or skills does this role require?

1. What role do you think your healthcare organization should play in addressing food insecurity in your patient population?

**Program Components—planning, development, implementation procedures, sustainability**

1. Please tell me about the major components of your program and who is responsible for executing each component.

***Probe:*** Screening and which tool is used

***Probe:*** Program Referral

***Probe:*** Program Enrollment

***Probe:*** Program Delivery

1. How did your organization decide to implement these components and who would be responsible for which component?

***Probe:*** How were program partners established?

**Organizational Support**

1. How are frontline providers trained or educated to execute the program?

***Probe:*** Individuals that oversee and provides training

***Probe:*** Ongoing consultation or technical support

***Probe:*** Pretest/posttest assessment of knowledge and skills

***Probe:*** Performance assessments

***Probe:*** Fidelity assessments—implementing the program as intended

1. What types of systems, policies or procedures have been implemented to support program implementation?

***Probe:*** EHR integration, what software system, who oversees this process, who provides technical support

***Probe:*** Integration into workflow or caseload management

***Probe:*** Data sharing procedures with external stakeholders

***Probe:*** Problem solving procedures within your organization and with external stakeholders

***Probe:*** Systems wide communications procedures

1. How does your leadership support program implementation?

***Probe:*** Culture change and motivation

***Probe:*** Technical support

***Probe:*** Content expertise

***Probe:*** Problem solving strategies

***Probe:*** Funding

***Probe:*** Communication

1. How does your organization measure program outcomes?
2. Overall, how effective do you think the program is?

***Probe:*** Number of patients screened

***Probe:*** Number of patients that participate in the program or receive services

***Probe:*** Number of patients that change behavior

***Probe:*** Addresses food insecurity

***Probe:*** Disease management

1. What do you consider are the strengths and limitations of the program?
2. What are major barriers to program implementation?
3. What are critical facilitators during program implementation?
4. Is there anything else you wish to share with me today about the program?

***Probe:***  program procedures, meeting notes discussing implementation processes

**Frontline Provider Interview Guide**

**INTRODUCTION:**

I’m conducting interviews with frontline providers from your organization to learn more about your experience with program implementation. I want to learn more about your role, program procedures, as well as barriers and facilitators you have encountered. The interview will be used to understand your experience with program implementation. Your participation in this interview is important because it will inform the development of program guidelines that may be distributed to other healthcare organizations interested in implementing a similar initiative.

Do you have any questions before we begin?

1. How would you define food insecurity or low food security?
2. What is your role or job title in this healthcare organization?
3. What is the name of the initiative we are discussing today and how long has your program been operating?
4. What is your role in implementing the (Program Name)?
5. How were you selected for this role?

***Probe:*** Were you assigned be senior staff, did you volunteer, were you nominated?

***Probe:*** What knowledge or skills does this role require?

1. What role do you think your healthcare organization should play in addressing food insecurity in your patient population?

**Screening**

1. Who is screened for food insecurity?

***Probe:*** What are the eligibility requirements or is everyone screened for food insecurity?

1. How does screening occur? Walk me through the screening process.

***Probe:*** Where does screening occur?

***Probe:*** What screening tool do you use?

***Probe:*** Is screening integrated into EHR?

***Probe:*** At what point in time during the patient visit does screening occur?

***Probe:*** How long does it take?

1. What do you think of this process?
2. Have you encountered any major barriers to this process?

***Probe:*** appropriate skills, knowledge, time, technical issues with systems and data management

1. What has helped support your adoption of screening practice behaviors?

***Probe:*** Training/knowledge/education, system support, data management processes, communication with managers

**Referral and Enrollment Process**

1. How do patients receive information about the food access service and enroll in the program? Walk me through this process.

***Probe:*** onsite or offsite

1. Overall, how effective do you think the program is?

***Probe:*** Number of patients screened

***Probe:*** Number of patients that participate in the program or receive services

***Probe:*** Number of patients that change behavior

***Probe:*** Addresses food insecurity

***Probe:*** Disease management

1. Is there anything else you want to share with me about the program?

***Probe:*** program screening and referral sheets, procedural guidelines, meeting notes and communication notes or email
