# Supplementary material for: An embedded multiple case study: using CFIR to map clinical food security screening constructs for the development of primary care practice guidelines
Source: BMC Public Health. 2022 Jan 14;22:97. doi: 10.1186/s12889-021-12407-y (PMC8758892; doi:10.1186/s12889-021-12407-y)
Supplement: Supplementary file 2 — Additional file 2. [file 12889_2021_12407_MOESM2_ESM.docx]

| Study Codebook | | | |
| --- | --- | --- | --- |
|  | **Construct 1: Intervention Characteristics** |  |  |
|  | ***Code*** | ***Category*** | ***Definition*** |
| a. | Source | Intervention Source | **Definition/Statements** Perception of key stakeholders about whether the intervention is externally or internally well-developed.  Include statements about the quality of current intervention design. |
| c. | Adaptability | Adaptability | **Definition/Statements** The degree to which an intervention can be adapted, tailored, refined or reinvented to meet local needs.  Include statements about adaptability of the intervention to the specific context of each clinic. |
| f. | Complexity | Complexity | **Definition/Statements** Perceived difficulty of implementation,  Include statements about duration, scope, disruptiveness, number of steps to implement. |
| g. | Design | Design/Program Elements | **Definition/Statements** Perception about how the intervention is designed.  Include statements about intervention design, specific program elements, intended program model--how things are supposed to work. |
|  |  | i. FI screening | **Definition/Statements** Any mention of the process for food insecurity screening, using validated screening questions. |
|  |  | (1) Too infrequent | **Definition/Statements**  This code refers to participant discussion about the inability to track and follow up with patients after initial screening due to the infrequency of routine FI screening that was intended by the program design. |
|  |  | (2) Ineffective | **Definition/Statements**  This code refers to participant discussion of food insecure patients that are not identified as food insecure during the screening process. |
|  |  | (3) Stigma | **Definition/Statements**  This code refers to any mention of patient discomfort to respond reliably to food insecurity questions during screening. |
|  |  | (4) Provider effectiveness | **Definition/Statements**  This code refers to any mention of doctor revealing food insecurity in patient that originally screened negative. |
|  |  | (5) EMR | **Definition/Statements** This code refers to any mention of using the EMR system for FI screening. |
|  |  | (6) Paper | **Definition/Statements** This code refers to any mention of using a paper format for FI screening. |
|  |  | ii. Referral | **Definition/Statements** Referral food distribution program. |
|  |  | (1) No Referral | **Definition/Statements** This code refers to any mention of a food insecure patient that does not receive a referral to the food distribution program due to challenges experienced during the implementation process.  Include statements about FI patients that were not effectively identified during screening or during conversations with doctor. |
|  |  | iii. Patient Education | **Definition/Statements** Any type of education/referral/enrollment in other, supporting resources, including local pantries, housing or job security programs. |
|  |  | (1) Lack of time | **Definition/Statements** This code refers to any mention of doctors' lack of time to talk to patients about food access resource list |
|  |  | (2) Alternative staff | **Definition/Statements** This code refers to the role SW's, clinic managers and front desk staff take to support providers' role in program implementation. |
|  |  | iv. Enrollment | Enrollment in SNAP and other federal benefits programs. |
|  |  | (1) Lack of time | **Definition/Statements** This code refers to any mention of the inability to enroll patients in SNAP due lack of staff time. |
|  |  | (2) Lack of training | **Definition/Statements** This code refers to any mention of the inability of staff to enroll patients in SNAP due to a lack of training to use online enrollment processes effectively. |
|  |  | (3) Patient disinterest | **Definition/Statements** This code refers to any mention of patients’ lack of interest to enroll in SNAP benefits. |
|  |  | v. Program promotion | **Definition/Statements** Any mention of program promotion or marketing to patients, reminders to increase participation including signage, phone calls, emails, word of mouth. |
|  |  | (1) Lack of time | **Definition/Statements** This code refers to any mention of the inability of staff to complete phone call reminders for program participation because of the lack of time |
|  |  | (2) Alternative method | **Definition/Statements** This code refers to any mention of alternative methods to reach patients to educated them about the program that deviates from the intended method for program promotion/marketing. |
|  |  | vi. Food distribution | **Definition/Statements** Any mention of food distribution processes. |
|  |  | (1) Lack of Space | **Definition/Statements** This code refers to any mention of the lack of space inside the clinic to distribute food to patients. |
|  |  | (2) Too infrequent | **Definition/Statements** This code refers to any mention of food distribution as a result of the program design that does not occur often enough to meet patient needs. |
|  |  | (3) Inaccessible | **Definition/Statements** This code refers to any mention of patient limited access to food distribution due to transportation or scheduling issues. |
|  |  | (4) Mobile food pantry/truck | **Definition/Statements** This code refers to any mention of the use of the mobile food truck service provided by the local food bank partner for Program A. |
|  |  | (5) Produce prescription program | **Definition/Statements** This code refers to any mention of the produce prescription program service provided by the urban garden collective for Program B. Also included with distribution is a training module for staff to provide nutrition education and cooking demonstrations to FI patients. |
|  |  | vii. Program evaluation | **Definition/Statements** Assessment of program outcomes, which includes change or no change observed in health outcomes, behavior change, dietary health, access to healthy food, patient participation rates as a result of patient participation in the screening and the food distribution elements of the program. |
|  |  | (1) Lack of EMR | **Definition/Statements** This code refers to participants expressing limited ability to use the EMR system for program evaluation. |
|  |  | (2) Patient participation rates | **Definition/Statements** This code refers to mention of ability to collect participation rates for program evaluation. |
| h.. | Cost | Program Cost | **Definition/Statements** Costs of the intervention and costs associated with implementing the intervention.  Include statements about program funding to pay for program elements, staff needs and additional resources. |
|  |  | i. Food distribution | **Definition/Statements**  This code refers to any mention of the cost of food distribution to meet program objectives. |
|  | **Construct 2. Outer Setting** |  |  |
|  | ***Code*** | ***Category*** | ***Definition, Inclusion Criteria*** |
| j. | Cosmopolitanism | Cosmopolitanism | **Definition/Statements**  The degree to which an organization is networked or partnered with multisector external organizations that support the intervention through capacity, resources and expertise.  Include statements about referrals to organizations that provide local food pantries, job services, housing, etc. that are tied to food insecurity. |
| l. | External Pressure | External Recommendations and Evidence | **Definition/Statements** External policy, research and recommendations (governmental or other central entity), benchmark reporting or community needs assessment that support the need for the intervention.  Include statements that mention the Community Health Needs Assessment (CHNA), Nat’l FI screening and referral policy recommendations or any other needs assessments conducted by external organizations or agencies. |
|  | **Construct 3:**  **Inner setting** |  |  |
|  | ***Code*** | ***Category*** | ***Definition, Inclusion Criteria*** |
| j**.** | Structural | Structural Characteristics | **Definition/Statements**  The structural maturity and size of an organization; infrastructure and physical space to support the intervention.  Include statements about the organization’s infrastructure to support the intervention, including hours of operation, location, EMR, number of and appropriate type of staff/implementation actors to support the program; frequency of staff turnover/physical space to allow for intervention to run smoothly and consistently. |
|  |  | i. Systems level | **Definition/Statements** This code refers to the organizational level where the program lives, which includes EMR infrastructure, funding/resources, culture and climate for program implementation. |
|  |  | ii. Regional level | **Definition/Statements** This code refers to the regional cluster of primary care clinics within an organization that is overseen by one Regional Director. |
|  |  | iii. Department level | **Definition/Statements** This code refers to departmental clusters of primary care clinics overseen by one Department Chair where program implementation occurs. |
|  |  | iv. Clinic level | **Definition/Statements** Each primary clinic within an organization that is overseen by one Clinic Manager or Clinic Coordinator where program implementation occurs. |
|  |  | v. EMR usability | **Definition/Statements** This code refers to how well the EMR system fits with intended program design and implementation. |
| k. | Communication | Networks and Communications | **Definition/Statements:** The nature and quality of formal and informal communications within an organization about program planning, execution reaching implementation goals and progress in meeting those goals; distribution of responsibilities.  Include statements about staff communicate with one another about program implementation; roles and responsibilities; how the quality of internal communication affects program implementation. This can include any mention of emails, internal memos, meetings, signs, etc. |
| l. | Culture | Culture | **Definition/Statements:** Norms, values and basic assumptions of a given organization and its staff that reflect the organization mission statement,  Include statements about whether the culture of the organization supports the intervention; does the intervention fit into the organization’s overall mission for patient care? |
| m. | Climate | Implementation Climate | **Definition/Statements:** The absorptive capacity for change; relative priority and shared perception of the importance of the intervention within the system  Include statements about whether the organization makes space to accept the program as a priority and a part of day-to-day activities. |
| n. | Readiness | Readiness for Implementation | **Definition/Statements:** Tangible and immediate indicators that an organization has prepared to implement the program; organization access of information and knowledge about program implementation  Include statements about assigned staff roles; dedicated time and space for screening, education and food distribution to follow through with the program as planned. |
|  | **Construct 4.**  **Characteristics of Individuals** |  |  |
|  | ***Code*** | ***Topic*** |  |
| o. | Knowledge | Knowledge and beliefs about the intervention | **Definition/Statements:** Individuals’ knowledge placed on the intervention as well as familiarity with facts, truths and principles related to the intervention.  Include statements about the individuals that implement the program that reflect their knowledge about FI, the patients they serve, implementation processes, goal of the program. |
| p. | Efficacy | Efficacy | **Definition/Statements:** Perception of own or staff capabilities to execute courses of action to achieve implementation goals.  Include statements individuals make about their own or others’ abilities to execute the intervention. Include statements about factors that affect how an individual implements the program, including time-management, training, etc. |
| r. | Identification | Individual Identification with Organization | **Definition/Statements:** A broad construct related to how individuals perceive or are perceived within the organization and their relationship and degree of commitment to implementation responsibilities.  Include statements about individuals’ roles within the organization and their responsibility for program implementation; if they feel their main job responsibilities take priority before program responsibilities. Do they feel they are responsible for program implementation or do they believe it is someone else’s job? How do others perceive their role in the organization and program implementation? |
| s. | Skills | Skills | **Definition/Statements:** Intellectual ability, competence, capacity, communication skills, empathy, ability to read patient needs that contribute to implementing the program as planned.  Include statements about patient-provider communication (language and cultural understanding) during screening, education and food distribution; ability to enroll in SNAP and WIC, provide additional resources for FI patients; cooking demos, recipes; ability to make patients feel comfortable. |
|  | **Construct 5. Process** |  |  |
|  | ***Code*** | ***Topic*** | ***Definition, Inclusion Criteria*** |
| t. | Planning | Planning | **Definition/Statements:** The degree to which a scheme or method of behavior and tasks for implementing an intervention are developed in advance and the quality of those schemes or methods (including marketing and promotion; planning for unforeseen barriers and challenges).  Include statements about the process of program development, education/training for implementation, as well as collaborative approach with food partner. |
| u. | Engaging | Engaging | **Definition/Statements:** Involving appropriate individuals in the ongoing implementation and problem solving 1. Opinion leaders, 2. formally appointed internal implementation leaders, 3. champions, 4. external change agents and community stakeholders.  Include statements about leadership engagement for effective implementation, external businesses and stakeholders for parking |
| v. | Fidelity | Fidelity | **Definition/Statements:** Carrying out or accomplishing the implementation according to plan.  Include statements about program execution as planned. |
|  | Executing unplanned | Changes to improve processes | **Definition/Statements:** Evolving implementation processes that deviate from the original implementation plan that results in improved implementation to meet patient and provider needs.  Include statements about lessons learned and improvements made at the clinic level due to unforeseen events or needs. |
| w. | Reflecting | Reflecting | **Definition/Statements:** Ongoing feedback/process evaluation about the progress and quality of implementation.  Include statements regular debriefing about progress and experiences, including process evaluation. |
|  | **Construct 6. Patient needs** | Patient Needs | **Definition/Statements:**  The extent to which patient needs are accurately known and prioritized by the organization.  Include statements about whether patient food needs are met through the intervention and whether they have access to specific intervention services frequent enough; if they have reliable transportation; if the food is culturally acceptable; if staff communicate appropriately; patient comfort |
|  |  | i. Program improvements | **Definition/Statements:** The extent to which the implementation team makes changes to the intended program design and related implementation processes to meet patient needs.  Include statements about ongoing efforts to improve implementation processes to meet patient needs. |
